# Purinergic Receptor Stimulation Reduces Cytotoxic Edema and Brain Infarcts in Mouse Induced by Photothrombosis by Energizing Glial Mitochondria

**DOI:** 10.1371/journal.pone.0014401

**Published:** 2010-12-22

**Authors:** Wei Zheng, Lora Talley Watts, Deborah M. Holstein, Suresh I. Prajapati, Charles Keller, Eileen H. Grass, Christi A. Walter, James D. Lechleiter

**Affiliations:** 1 Department of Cellular and Structural Biology, University of Texas Health Science Center at San Antonio, San Antonio, Texas, United States of America; 2 Greenhey Children's Cancer Research Institute, University of Texas Health Science Center at San Antonio, San Antonio, Texas, United States of America; The Mental Health Research Institute of Victoria, Australia

## Abstract

Treatments to improve the neurological outcome of edema and cerebral ischemic stroke are severely limited. Here, we present the first *in vivo* single cell images of cortical mouse astrocytes documenting the impact of single vessel photothrombosis on cytotoxic edema and cerebral infarcts. The volume of astrocytes expressing green fluorescent protein (GFP) increased by over 600% within 3 hours of ischemia. The subsequent growth of cerebral infarcts was easily followed as the loss of GFP fluorescence as astrocytes lysed. Cytotoxic edema and the magnitude of ischemic lesions were significantly reduced by treatment with the purinergic ligand 2-methylthioladenosine 5′ diphosphate (2-MeSADP), an agonist with high specificity for the purinergic receptor type 1 isoform (P2Y_1_R). At 24 hours, cytotoxic edema in astrocytes was still apparent at the penumbra and preceded the cell lysis that defined the infarct. Delayed 2MeSADP treatment, 24 hours after the initial thrombosis, also significantly reduced cytotoxic edema and the continued growth of the brain infarction. Pharmacological and genetic evidence are presented indicating that 2MeSADP protection is mediated by enhanced astrocyte mitochondrial metabolism via increased inositol trisphosphate (IP_3_)-dependent Ca^2+^ release. We suggest that mitochondria play a critical role in astrocyte energy metabolism in the penumbra of ischemic lesions, where low ATP levels are widely accepted to be responsible for cytotoxic edema. Enhancement of this energy source could have similar protective benefits for a wide range of brain injuries.

## Introduction

Brain edema and infarctions result from multiple insults, but are typically preceded by cytotoxic edema [1], [2], [3]. Cell swelling is most prominent in astrocytes and appears to be initiated by Na+ accumulation due to failure of energy dependent ion extrusion [2]. ATP levels are depleted since oxidative phosphorylation is abrogated during ischemia or hypoxia [4]. Brain swelling is generally attributed to vasogenic edema caused by breakdown of the blood-brain barrier (BBB) and accumulation of extracellular water [1], [4]. Astrocyte necrosis subsequent to osmotic expansion damages adjacent tissue and expands the infarct by secondary mechanisms [4], [5], [6]. The expanding infarction core with a dynamic peri-infarct penumbra is not yet irreversibly injured and consequently, serves as the primary target for brain protection strategies [7]. Astrocytes are known to play a crucial role in supporting and protecting neuronal function as well as modulating brain energy metabolism [8], [9], [10], [11]. It is less well appreciated that astrocyte mitochondria themselves play an important role in these brain functions [8], [12]. Consequently, enhancing mitochondrial metabolism in astrocytes is a relatively unexplored strategy to decrease edema and preserve the penumbra [13]. Recent nuclear magnetic resonance (NMR) spectroscopic studies detecting astrocyte-specific mitochondrial acetate metabolism in viable and nonviable penumbra were predictive of neuronal survival [14]. In addition, inhibition of glial mitochondria is known to increase astrocyte swelling and lead to oncotic cell death [15], [16]. Taken together, these reports are consistent with the hypothesis that glial mitochondrial metabolism is a key determinant of cytotoxic edema, necrosis and neuronal survival during cerebral ischemic stroke.

The metabotropic IP_3_-mediated intracellular Ca^2+^ signaling pathway provides a very efficient mechanism to rapidly increase mitochondrial metabolism by activation of Ca^2+^ sensitive matrix dehydrogenases [17], [18]. This pathway increases the production of intracellular ATP up to ten-fold faster than stimulation by feedback from ADP/ATP pools [19]. IP_3_-mediated Ca^2+^ release in astrocytes stimulated by a variety of G-protein coupled receptors has been well documented [20], [21]. We recently demonstrated that stimulation of G-protein coupled purinergic receptors (P2Y_1_Rs) increased Ca^2+^ sensitive mitochondrial metabolism in astrocytes [22]. We also found that the resistance of astrocytes and co-cultured neurons to oxidative stress was enhanced in a mitochondrial energy dependent manner [22]. Here, we have focused our investigation on the potential protective role of P2Y_1_R signaling *in vivo*, using a Rose Bengal (RB)–induced photothrombotic model of focal cerebral ischemia [23]. We present evidence demonstrating that P2Y_1_R stimulation reduces astrocyte cytotoxic edema, necrosis, and neuronal cell death in mice with RB-induced brain lesions. Pharmacological and genetic evidence indicate that P2Y_1_R-enhanced protection is dependent on stimulation of astrocyte mitochondrial metabolism. These data suggest that astrocyte mitochondria are a key energy source in the post-ischemic penumbra, which can be stimulated by IP_3_-mediated intracellular Ca^2+^ release to significantly improve the neurological outcome subsequent to brain injuries.

## Results

### P2Y_1_R *Agonist 2MeSADP Decreases RB-induced Cerebral Infarcts in Mouse*

**Figure 1:**
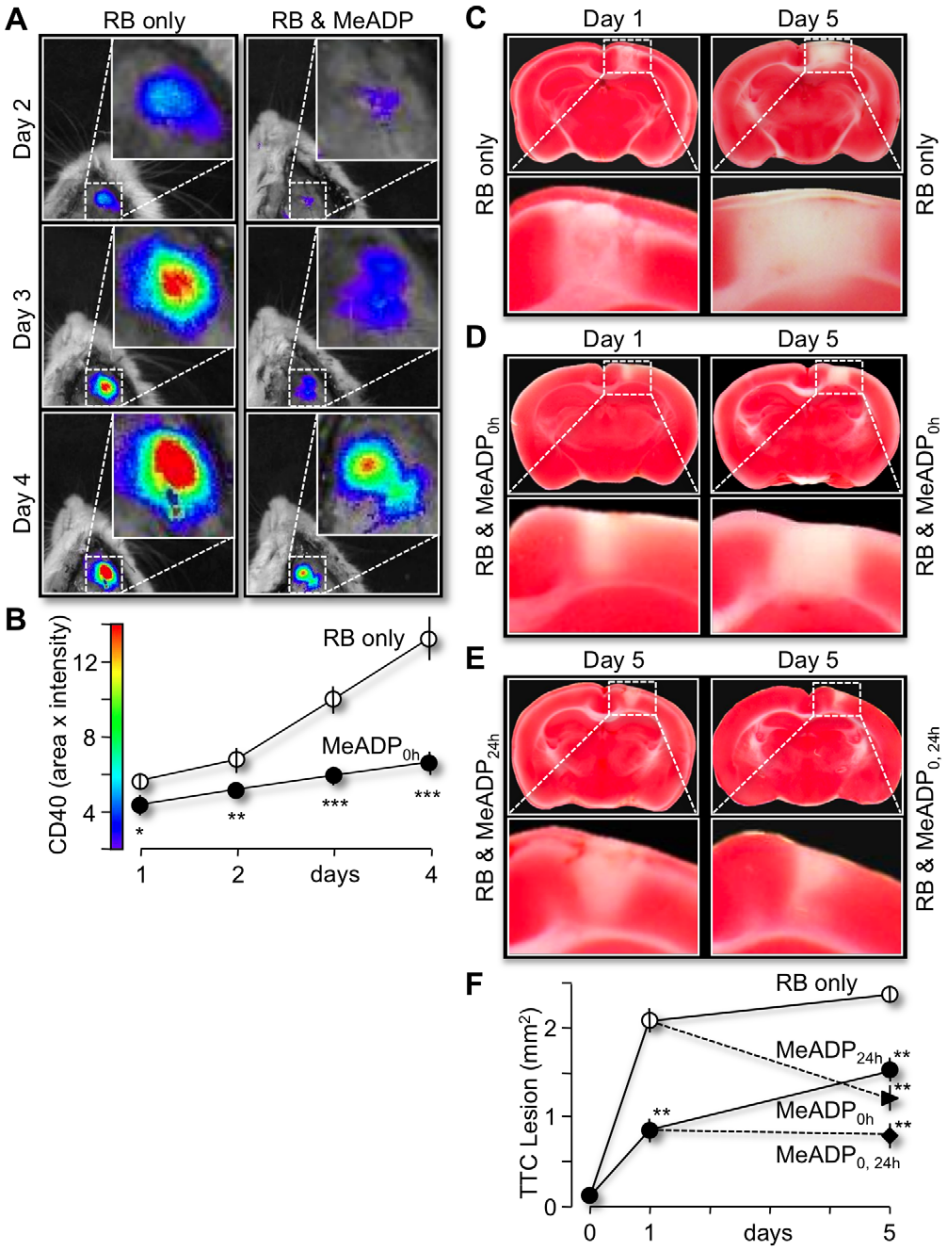
2-MeSADP enhanced protection of Rose Bengal (RB)-induced cerebral lesions in mouse cortex. (**A**) Fluorescent images of RB-induced cerebral infarcts of anesthetized mice initially treated with RB (left panels) or RB plus 2-MeSADP (right panels) on days 2, 3 and 4 after the initial photothrombosis. RB (20 µg/ml) was illuminated at 543 nm through the cranium with a 10× objective for 15 minutes on day 0. (**B**) Line plots of average intensity of the RB-induced infarcts times their area are presented for each group of animals (n = 4 pairs, *p<0.05, **p<0.01, ***p<0.001). Cerebral infarcts for both animal models were fluorescently labeled with an allophycocyanin (APC)-CD40 antibody 16 hours earlier by tail vein injection. Images were acquired on a Xenogen IVIS 200 fluorescent imaging system. (**C–E**) Images of coronal brain slices obtained from mice with photothrombotic-induced cerebral infarcts on day 0 as described in Figure. 1. Brain slices were stained for live mitochondria using 2,3,5-triphenyltetrazolium chloride (TTC) staining (Li et al., 1997) on day 1 or Day 5 as indicated. Necrotic tissue is identified in each panel (dashed rectangles) as the absence of red staining, indicating non-respirating mitochondria. Note the significant reduction in lesion area when 2-MeSADP is injected either at day 0 (B), at day 1 (C, left panels) or at days 0 and 1 (C, right panels). (**F**) Graph of average size of lesions at the indicated days. Means are calculated from at least 5 mice for each point. **p<0.01.

To induce an acute cerebral infarction, a small region in the mouse parietal cortex containing multiple blood vessels was clotted by Rose Bengal (RB) induced photothrombosis (Fig. S1). In brief, an incision was made in the animal scalp under anesthesia to expose the translucent skull, which was subsequently thinned and irradiated with green light (543 nm) for ∼10 minutes after tail-vein injecting the photosensitive RB dye. When excited for prolonged periods, RB generates singlet oxygen molecules that locally damage the blood vessel walls and trigger thrombosis [23]. We utilized CD40 as a molecular marker to visualize the RB-induced cerebral infarctions. CD40 is a membrane protein of the tumor necrosis factor receptor family that has been reported to be an early important contributor to tissue necrosis during acute ischemic infarction [24]. Fluorescently labeled CD40 antibody (APC-anti-mouse CD40) was tail-vein injected 16 hours prior to imaging the necrotic tissue with a Xenogen IVIS 200 fluorescence system. As expected for an expanding cerebral ischemic lesion, the average magnitude of CD40 fluorescence (area×intensity) increased from 5.60±0.41×10^8^ photons/s on day 1 to 6.72±0.68×10^8^ photons/s on day 2, to 9.97±0.75×10^8^ photons/s on day 3 and to 1.33±1.20×10^9^ photons/s on day 4 post-photothrombosis (n = 5, Fig. 1A, B).

The same experimental protocol was followed to test the impact of P2Y_1_R stimulation on RB-induced brain infarctions. Initially, the P2Y_1_R agonist 2MeSADP was tail-vein injected in conjunction with RB prior to photothrombosis. We found that the presence of 2MeSADP (100µM, 0.1ml) significantly reduced the average size and intensity of CD40 signals to 4.35±0.58×10^8^ photons/s on day 1, to 5.18±0.22×10^8^ photons/s on day 2, to 5.91±0.51×10^8^ photons/s on day 3, and to 6.60±0.64×10^8^ photons/s on day 4 post-photothrombosis when compared to control mice (n = 5, Fig. 1A, B). No significant protective effects were observed at lower concentrations of 2MeSADP (10 µM, 0.1ml) and administration of higher doses of 2MeSADP (1000 µM, 0.1ml) caused respiratory inhibition and neurotoxocicity (see Methods). A parallel set of experiments was performed to directly measure the size of RB-induced lesions in brain slices stained with the vital dye 2,3,5-Tripheyltetrazolium chloride (TTC). TTC is a colorless dye that stains healthy brain tissue red when reduced by the mitochondrial enzyme succinyl dehydrogenase [25]. The absence of staining in necrotic tissue is then used to define the area of a brain infarction. As observed with CD40 fluorescence labeling, the mean area of the RB-induced lesion increased from 2.07 mm^3^±0.24 (n = 8) on day 1 post-photothrombosis to 2.32±0.23 on day 5 in control animals (Fig. 1F). When 2MeSADP (100 µM) was included with the RB dye, the average size of the brain lesions was significantly reduced to 0.86 mm^3^±0.19 (n = 9; p<0.001) on day 1 and to 1.54mm^3^±0.23 (n = 11, p<0.001) on day 5 (Fig. 1C–F). In a separate group of experimental mice, 2MeSADP was injected a second time (boosted), 24 hours after the initial injection and photothrombosis. These mice exhibited the largest reduction in lesion size at day 5, 0.77 mm^3^±0.24 (n = 7, Fig. 1E). Finally, we tested the efficacy of 2MeSADP to reduce lesions when treatment was delayed 24 hours after the initial photothrombosis. Remarkably, we found that the average lesion size of this group at day 5 was also significantly reduced to 1.24±0.26 (n = 6, p<0.05) compared to untreated controls. In fact, the efficacy of 2MeSADP introduced 24 hours after the initial photothrombosis to reduce lesions was not significantly different from that of 2MeSADP injected with RB dye (Fig. 1D–F).

**Figure 2:**
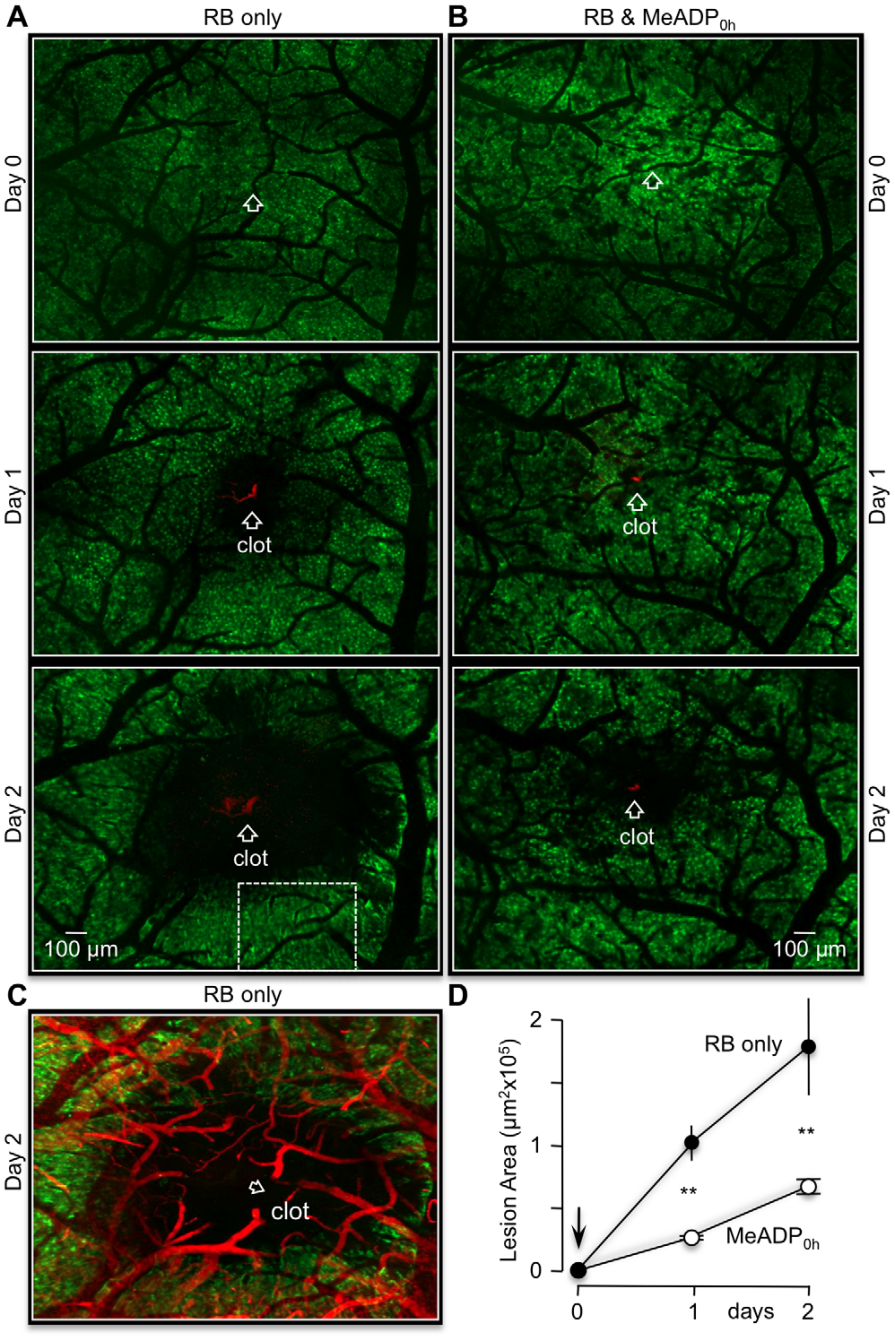
2-MeSADP treatment decreases *in vivo* lesions induced by single vessel photothrombosis. (**A**) Mosaic reconstruction of approximately 30 imaging fields of the parietal cortex of live transgenic mice expressing GFP in astrocytes (GFAP-GFP). The same cortical region was imaged at day 0 (pre-photothrombosis) and at 24, 48 hours post-photothrombosis. RB was tail vein injected and irradiated (543 nm) at the single blood vessel indicated by the open white arrows, triggering photothrombosis at 0 hours (labeled as clot in 48 hours). Astrocyte cell lysis at 24 and 48 hours is apparent by the absence of GFP fluorescence. White dashed rectangle indicates region that is presented at higher magnification in Fig. S4A. (**B**) Experimental mouse that was co-injected with RB and the P2Y_1_-R ligand, 2-MeSADP. Note the dramatic reduction in the stroke lesion at 24 and 48 hours compared to panels in (A). (**C**) Mosaic image of day 2 panel in (A) overlaid with mosaic image of rhodamine-dextran filled vasculature. (**D**) Line plots of the average lesion size of 5 control mice (RB only) and 5 experimental mice (RB+ 2-MeSADP). **p<0.01.

**Figure 3:**
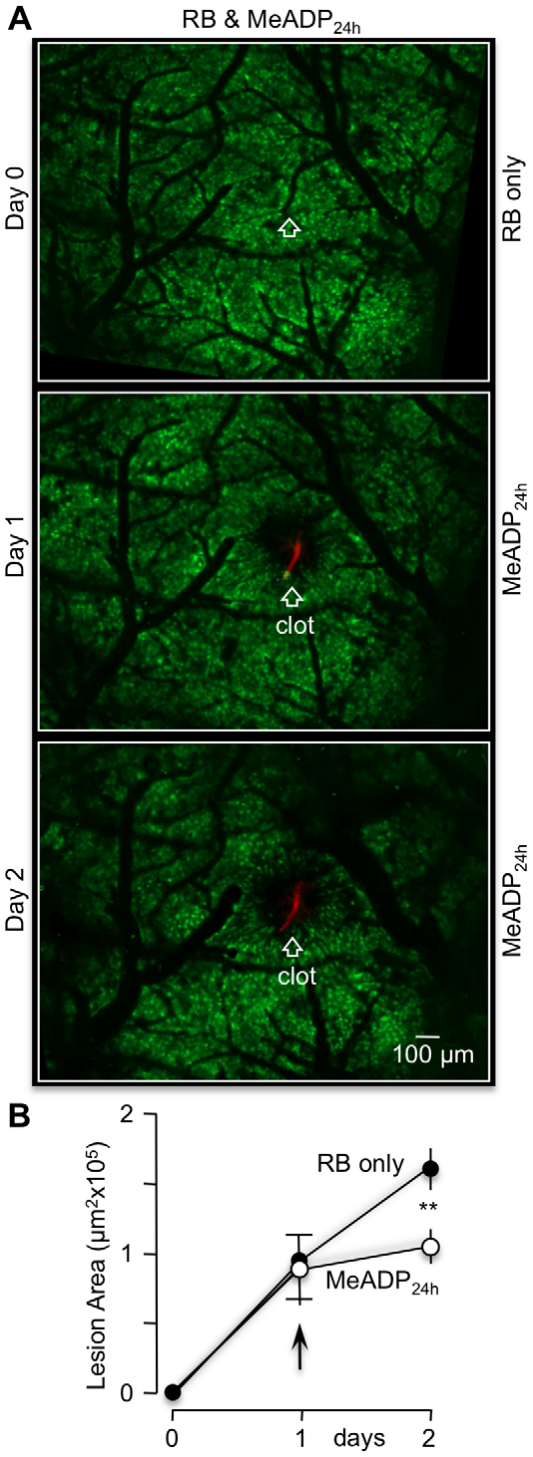
24 hour delayed 2-MeSADP treatment decreases the size of cortical lesions induced by single vessel photothrombosis. (**A**) Mosaic reconstruction of the parietal cortex from GFAP-GFP transgenic mice imaged at day 0 (pre-photothrombosis) and at 24, 48 hours post-photothrombosis. RB was tail vein injected and a single vessel indicated by open white arrow was irradiated (543 nm). At day 1 post-thrombosis, the P2Y_1_-R ligand, 2-MeSADP was tail vein injected. Note the significant slowing of infarct progression at day 2 (lower panel). (**B**) Line plot summary of 5 control (RB only) and 5 experimental mice (RB+2-MeSADP). **p<0.01.

To further investigate the impact of P2Y_1_R stimulation on cerebral ischemic lesions, we performed a series of *in vivo* imaging experiments utilizing transgenic mice expressing green fluorescent protein (GFP) in astrocytes (FVB/N-Tg(GFAPGFP)14Mes/J, JAX® Mice). The cell bodies of individual astrocytes were easily imaged with a confocal microscope through a thinned skull window in the parietal cortex of these mice (Fig. S1 and S2). When a single bolus of RB was tail-vein injected, a luminal increase in blood vessel fluorescence was transiently observed in the imaging window. Single vessel photothrombosis could be induced by irradiating a zoomed region of an RB-filled arteriole with green light (543 nm) for ∼5 minutes (Fig. S1D). Large mosaic images of the cortex were then seamlessly reconstructed from ∼30 individual z-stacks to follow the effect of a single vessel clot. Twenty-four hours after photothrombosis, an infarction border could easily be identified by the absence (lysis) of GFP labeled astrocytes near the clotted single vessel. The clot itself was also easy to identify by the red fluorescence of trapped RB dye (Fig. 2A). We found that the average size of an RB-induced lesion at 24 hours was 1.02±0.07 µm^2^×10^5^ (n = 5). This area increased to 2.14±0.22 µm ^2^×10^5^ (n = 5) by 48 hours. Pharmacological agents are known to leak into local brain tissue following blood clotting due to the disruption of the blood brain barrier (BBB). An increase in BBB permeability was observed in these preparations as leakage of RB dye into the extravascular space (Fig. S2). Extravasation of re-injected RB dye from a clotted arteriole was also obvious two days after the initial photothrombosis (Fig. S2). We took advantage of the BBB permeability breakdown to introduce P2Y_1_R agonist into the ischemic region. We determined that a tail-vein injection of 2MeSADP (100 µM) along with RB dye at the initial photothrombosis significantly decreased the stroke lesion size at 24 hours to 0.30±0.02 µm^2^×10^5^ (n = 5) and to 0.59±0.07 µm^2^×10^5^ (n = 5) at 48 hours (Fig. 2D). Moreover, tail-vein injections of 2MeSADP could again be delayed 24 hours after the initial photothrombosis and still significantly slow the progression of the lesion. Specifically, RB-induced lesions in both control and test mice were permitted to expand to 0.95±0.17×10^5^ µm^2^ (n = 3) and 0.89±0.12 µm^2^×10^5^ (n = 3) for 24 hours, respectively (Fig. 3). The average lesion size of mice subsequently injected with 2MeSADP was 0.88±0.15×10^5^ µm^2^ (n = 3) at 48 hours compared to 1.97±0.15×10^5^ µm^2^ (n = 3) for non-treated mice.

### P2Y_1_R stimulation Reduces RB-induced Ischemic Astrocyte Swelling and Necrosis

**Figure 4:**
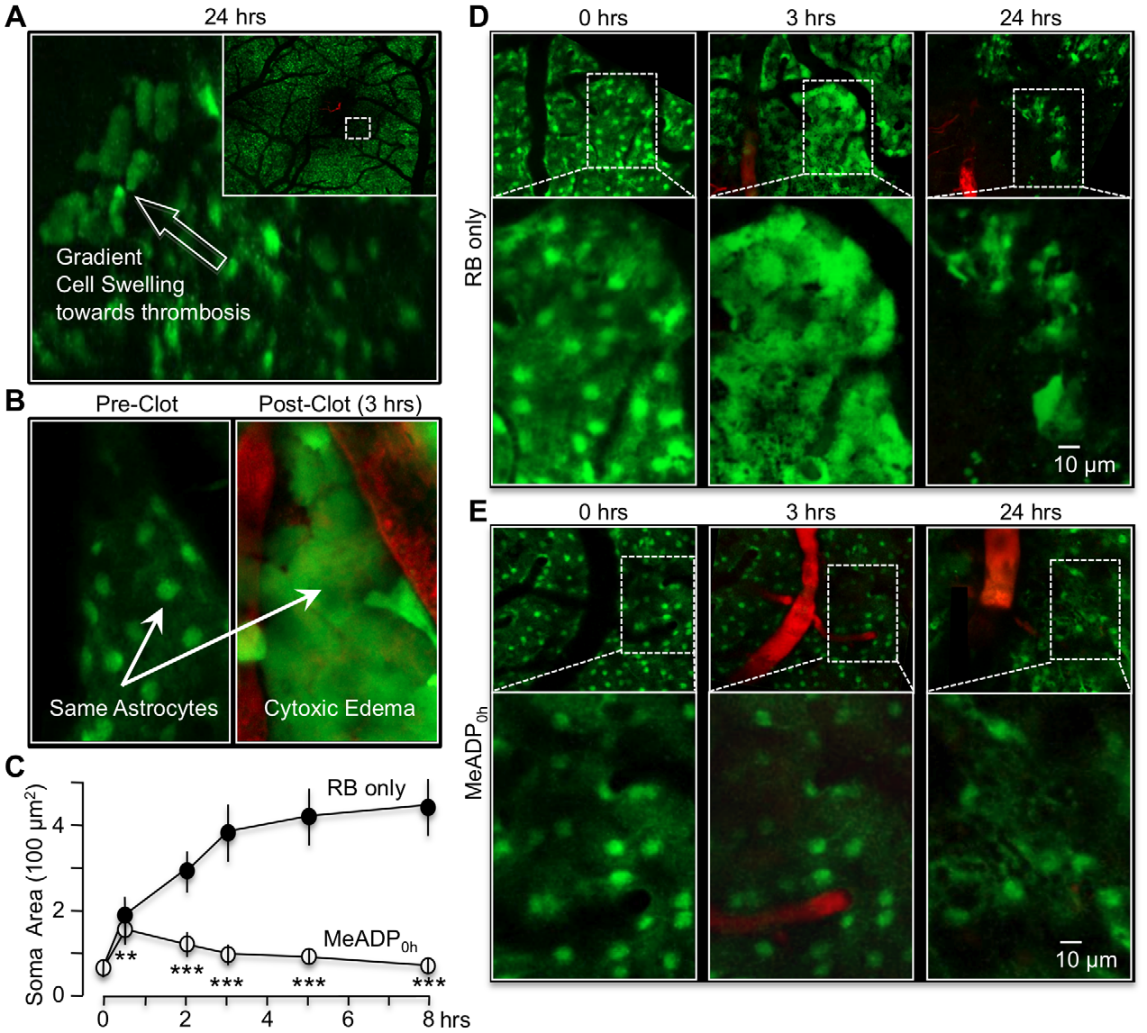
2-MeSADP treatment significantly reduces RB-induced post-ischemic cytotoxic swelling and necrosis of cortical astrocytes. (**A**) Confocal image at the boundary of an RB-induced lesion. The white arrow indicates a gradient of astrocyte swelling nearest the infarct 24 hours post-clot in a GFAP-GFP mouse. The insert shows a lower magnification mosaic image of cortex near the infarct, initially presented in Fig. 3A. Dashed rectangle corresponds to higher magnification image. (**B**) Rapid astrocyte swelling near RB-induced photothrombosis in GFAP-GFP transgenic mouse. The same region of the cortex was periodically imaged just prior to (0 min) and after ischemia at 30, 80, 140 min. The insert highlights a single swollen astrocyte. (**C**) Lineplot of average size of astrocyte somas near clot as a function of time. The swelling is close to maximum by 3 hours of the initial clot. Means +/− SD (n = 38 cells) pooled from 5 control RB only mice and 5 2-MeSADP treated mice. ** p<0.01 *** p<0.001 (**D**) High magnification images of cortical region near a blood vessel just prior to (0 hrs) and after photothombosis (3 and 24 hrs). Oncotic cell death (the absence of green fluorescence) is apparent at 24 hours post-thrombosis, although some astrocytes further from the lesion appear to have reversed swelling. (**E**) Comparable high magnification images near clotted vessel treated with 2-MeSADP. Note that astrocyte swelling proximal to the clotted vessel is absent 3 hours post-thrombosis and that many of these cells have not lysed even at 24 hours.

Astrocyte swelling and necrosis are considered major causes of tissue damage after focal brain ischemia [16]. Consistent with this etiology, we observed severely swollen astrocytes near the edge of RB-induced infarcts with a gradient of cell swelling towards thrombosis (Fig. 4A). Temporal imaging of perivascular astrocytes immediately after a single RB-induced photothrombosis revealed rapid swelling with the average size of the cell soma increasing over 6-fold within 3 hours, from 63±15 µm^2^ at rest to 382±61 µm^2^ (mean ± SD, n = 38 cells pooled from 5 mice, Fig. 4B, C). Swelling gradually spread outward from the ischemic core to adjacent tissue (Fig. 4A). By 24 hours, many of the swollen astrocytes had lysed (necrosis), although some of the cells distal to the clot exhibited reversible swelling (Fig. 4D, 6F). In contrast, astrocyte swelling in the presence of 2MeSADP post-photothrombosis was significantly reduced. At 3 hours, the average soma size was only 99±22 µm^2^, and at 24 hours, many of the astrocytes near the ischemic core had still not lysed (Fig. 4C, E). Neither laser illumination nor the RB dye by itself affected astrocyte swelling (data not shown). These data suggest that a key mechanism of action underlying 2MeSADP-mediated protection is its ability to reduce astrocyte swelling and necrosis.

### Functional Astrocyte Mitochondria are required for P2Y_1_R-mediated decreases in RB-induced Cerebral Infarcts

**Figure 5:**
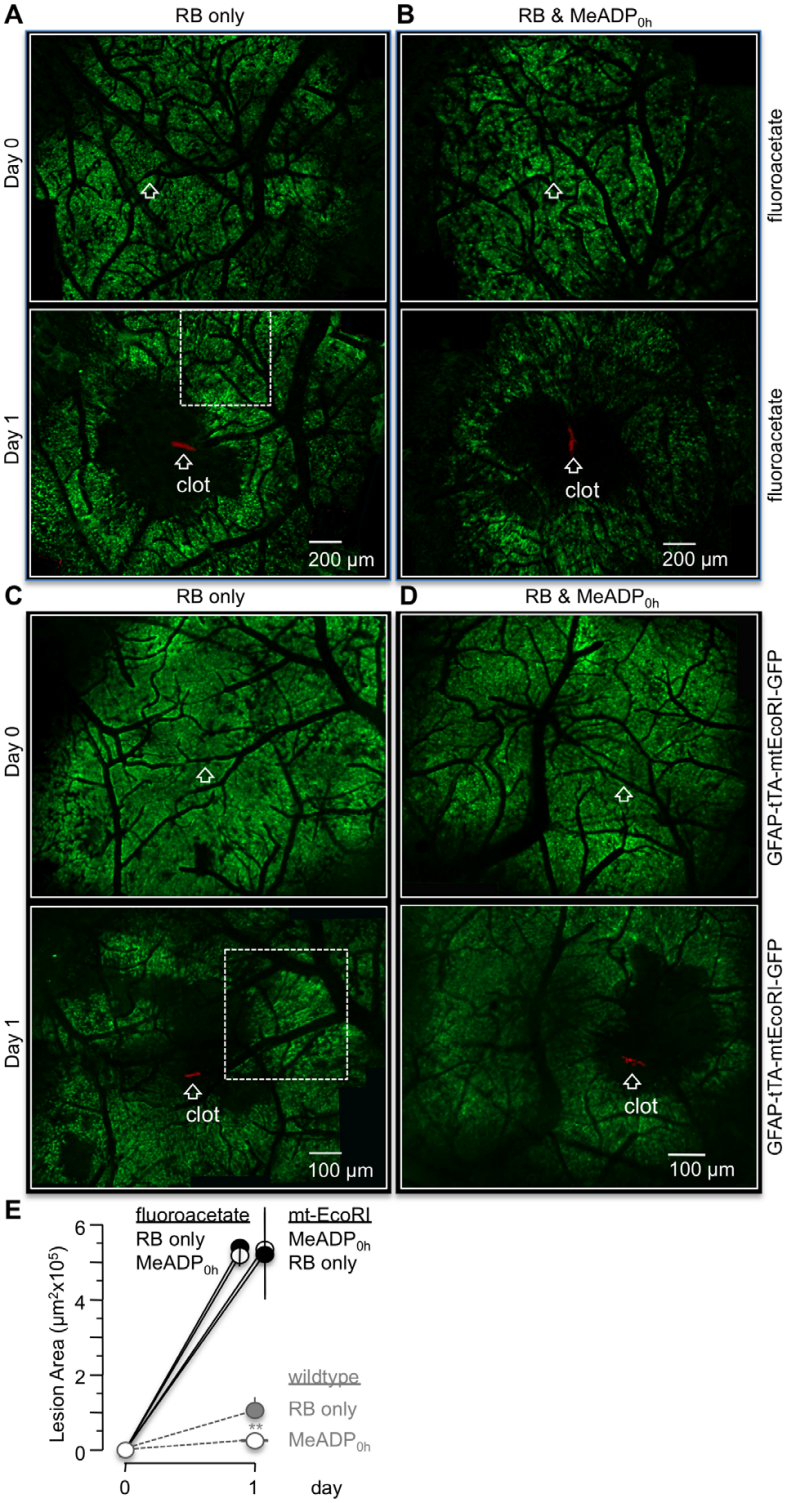
Disruption of astrocyte mitochondrial metabolism increases RB-induced lesions and blocks 2-MeSADP stimulated protection. (**A** and **B**) Mosaic image panels from the parietal cortex of fluoroacetate treated GFAP-GFP transgenic mice. Mice were imaged at day 0 (pre-photothrombosis) and at day 1 (post-photothrombosis) with (A) and without 2-MeSADP treatment (B). RB-induced photothrombosis is labeled as clot by white arrows. Fluoroacetate, the tricarboxylic acid (TCA) cycle toxin, was injected with RB. White dashed rectangle indicates region that is presented at higher magnification in Fig. 3B. (**C** and **D**) Mosaic image panels of GFAP-tTA-mtEcoRI-GFP mice imaged at day 0 (pre-photothrombosis) and day 2 (post-photothrombosis) with (D) or without (C) 2-MeSADP. White dashed rectangle indicates region that is presented at higher magnification in Fig. S3C. (**E**) Line plot showing average lesion sizes of fluoroacetate treated wildtype mice (3 RB only, 3 RB+2-MeSADP) and of mt-EcoRI expressing mice (3 RB only, 3 RB+2-MeSADP). Grayed symbols and lines are from wildtype data replotted from Fig. 2D for comparison. **p<0.01.

Earlier *in vitro* work from this laboratory demonstrated that P2Y_1_R-enhanced astrocyte and neuronal protection was dependent on astrocyte mitochondrial ATP production [22]. We employed a two-fold strategy to test the *in vivo* impact of astrocyte mitochondrial metabolism on P2Y_1_R enhanced protection. First, we pharmacologically disrupted astrocyte mitochondrial function by tail-vein injecting the tricarboxylic acid (TCA) cycle toxin, fluoroacetate (100 µM, 0.1ml), prior to inducing photothrombosis. Fluoroacetate is preferentially transported into astrocytes by monocarboxylic acid transporter isoforms (MCT1 and 4) that are not present in neurons [26]. When single vessels were clotted in the presence of fluoroacetate, the average infarction size at 24 hours was significantly increased to 5.19±0.28 µm^2^×10^5^ (n = 3) compared to non-treated controls (Fig. 5A, E). When 2MeSADP was co-injected with fluoroacetate, the average size of the RB-induced lesions at 24 hours was 5.41±0.14 µm^2^×10^5^ (n = 3), essentially unchanged compared to fluoroacetate only lesions (Fig. 5A, B, E). Our second strategy was to utilize a new transgenic mouse model that genetically disrupts mitochondrial function by expression of a mitochondrial targeted DNA restriction enzyme (EcoRI) under the doxycycline (dox) controlled transactivator protein (tTA^dox off^/mtEcoRI [27]). Control experiments demonstrated that in the presence of dox (no tTA expression), RB-induced lesions in GFAP-tTA-mtEcoRI mice were indistinguishable from wildtype mice (Fig. S3 A, B) and they were still reduced by 2MeSADP treatment (Fig. S3 C, D). We crossed this transgenic with two mouse lines that express GFP and tTA, respectively, under control of the astrocyte-specific transcriptional promoter GFAP (GFAP-GFP-tTA^dox off^-mtEcoRI). Dox was removed from these mice for two weeks to degrade astrocyte mitochondrial DNA and disrupt mitochondrial function [27] (Figs. S4 and S5). Similar to our observations with fluoroacetate treated mice, we found that the average size of lesions in mice with degraded astrocyte mitochondrial DNA was significantly increased to 5.27±0.41×10^5^ µm^2^ (n = 3) compared to wildtype control mice (Fig. 5C, E). Additionally, the average size of the RB-induced lesions at 24 hours was essentially unchanged at 5.43±0.50×10^5^ µm^2^ (n = 3) when 2MeSADP was tail-vein injected (Fig. 5D, E). We concluded from these experiments that disruption of astrocyte mitochondrial function results in significantly larger RB-induced lesions and that protection with the P2Y_1_R agonist 2MeSADP is absent.

**Figure 6:**
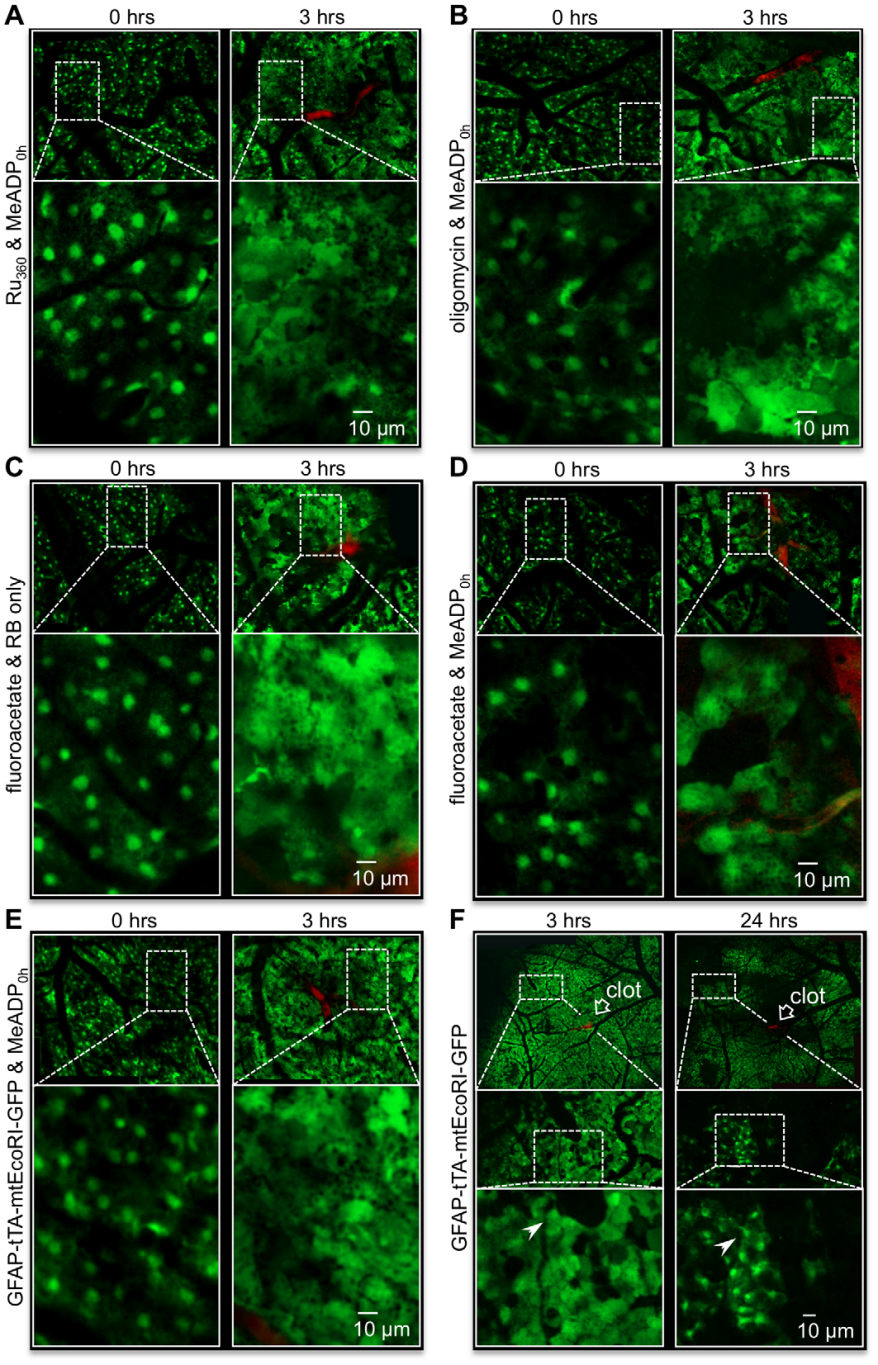
Disruption of mitochondrial function blocks the reduction of post-ischemic swelling by 2-MeSADP treatment in cortical astrocytes of GFAP-GFP mice. (**A**–**D**) High magnification confocal images of astroctyes before (0 hrs) and after (3 hrs) a single vessel photothrombosis in wildtype mice treated with either the mitochondrial Ca^2+^ uniporter inhibitor Ru_360_ (A), the mitochondrial ATPase inhibitor oligomycin (B) or the mitochondrial citric acid cycle inhibitor fluoroacetate (C and D). (**E**) Similar high magnification images obtained from GFAP-tTA-mtEcoRI-GFP mice. Note that significant swelling at 3 hours is observed in each case that mitochondrial function is disrupted even when treated with 2-MeSADP. (**F**) Confocal images of the cortical region of a GFAP-tTA-mtEcoRI-GFP mouse post-photothrombosis. Astrocytes at large distances (>500 µm) from the ischemic core exhibit severe swelling at 3 hours. At 24 hours post-thrombosis, significant necrosis is apparent from the lack of green fluorescence. However, some astrocytes distal to the clot still undergo reversible swelling. White arrowhead identifies the same astrocyte in each field.

We further tested whether disrupting mitochondrial function in astrocytes inhibited the ability of 2MeSADP treatment to reduce astrocyte swelling. Pharmacological inhibitors of mitochondrial Ca^2+^ uptake (Ruthenium 360), ATP synthesis (oligomycin) or the astrocyte TCA cycle (fluoroacetate) were co-injected with 2MeSADP. Each of these inhibitors completely blocked the ability of 2MeSADP to reduce astrocyte swelling as measured 3 hours post-photothrombosis (Fig. 6A–D). We also investigated the ability of 2MeSADP treatment to regulate astrocyte swelling in GFAP-GFP-tTAmtEcoRI mice that were removed from doxycycline for 2 weeks prior to RB-induced photothrombosis. Again, functional disruption of mitochondria in this astrocyte specific genetic model eliminated the ability of 2MeSADP to decrease astrocyte swelling as measured at the 3 hour time point post-photothrombosis (Fig. 6E, F). These data suggest that 2MeSADP-induced reduction of cytotoxic edema is dependent on astrocyte mitochondrial ATP production during ischemic brain injuries.

## Discussion

Astrocytes are known to play critical roles in neuronal survival subsequent to both ischemic injury and brain trauma [6], [28]. Our studies have extended these findings in three significant ways. First, we demonstrated that occlusion of single blood vessels by photothrombosis leads to rapid cytotoxic edema in astrocytes followed by a progressive cell lysis and brain infarction. Second, cell swelling, necrosis and brain infarcts were significantly reduced by treatment with the purinergic ligand 2MeSADP, which was effective when applied immediately as well as 24 hours after the initial thrombosis. Third, our evidence suggested that the mechanism by which 2MeSADP-reduced both cytotoxic edema and brain infarctions was dependent on astrocyte mitochondrial metabolism. This dependence on mitochondrial function is consistent with an accepted underlying cause of cell swelling, which is the failure of energy dependent ion extrusion due to abrogation of oxidative phosphorylation during hypoxia or ischemia [2], [4]. It is also interesting to note that 2MeSADP treatment 24 hours after the initial lesion resulted in a significant reduction the TTC defined lesion, which suggests that regions of reduced TTC staining (i.e. the penumbra) can be rescued by energizing astrocyte mitochondria.

Brain edema is generally separated into two components, an initial osmotic swelling of cells (cytotoxic edema) that depletes the extracellular space of Na+, Cl− and water, followed vasogenic edema, which appears to be primarily, but not exclusively responsible for brain swelling [1], [2], [6], [15], [29], [30]. The predominant component that we observed in response to photothrombosis was cytotoxic edema (Movie S1). Swelling appears to be tolerated by the unfolding of astrocyte membranes [31], [32]. Interestingly, the magnitude of astrocyte swelling, which we estimated by the measuring extent of GFP fluorescence, appeared to be significantly larger than measurements previously recorded in cell culture [31], [32] or *in vivo* [33]. This could be due to differences in our experimental conditions. Alternatively, our volume indicator, expression of GFP, may not always represent the total cell volume. In support of this, NMR measurements have indicated that the nucleus exhibits more bulk-like water properties than the cytosol [34]. Our pre-clot measurements of cell volume could represent a subvolume of the cell due to the restriction of GFP fluorescence to bulk water in the nuclear region. During cytotoxic edema, the cytosolic increase in bulk water could then provide an expanded environment into which GFP can freely diffuse. We also noticed that some of the astrocytes near the edge of our infarctions underwent reversible swelling, averting cell death at least temporarily (Fig. 6F, Movie S2). The reversibility of astrocyte swelling could be related to a simple increase in energy dependent ion extrusion. An additional mechanism of action could be stimulation of the regulatory volume decrease (RVD) [15]. Studies *in vitro* have shown RVD is energy-dependent in astrocytes [35], [36].

Several studies have suggested roles for P2Y_1_Rs in the regulation of astrocyte swelling [37], [38], [39], [40]. Darby and co-workers reported that autocrine release of ATP in cultured astrocytes leads to activation of P2Y_1_Rs, which in turn stimulates the RVD [40]. On the other hand, Mongin and Kimelberg found no requirement for autocrine ATP release for swelling induced activation of volume regulation ion channels [38]. Uckermann and co-workers reported that purinergic mechanism of glial swelling inhibition was mediated by activation of A1 receptors, which activated K+ and Cl− channels via protein kinase A [37]. Additionally, Fujita and colleagues found that P2Y_1_R signaling enhanced neuroprotection by stimulating release of IL-6 in hippocampal cultures [39]. Our data expand the protective role of P2Y_1_Rs during cytotoxic edema and suggest that 2MeSADP-decreased swelling is mediated by stimulation of the IP_3_-Ca^2+^ signaling pathway, ultimately increasing intracellular ATP production in mitochondria. Increases in astrocyte Ca^2+^ have also been reported to dilate arterioles via cyclooxygenase mediated formation of prostaglandin E2 [41], [42], [43]. This mechanism of action would be expected to increase perfusion in the penumbra, and hence, could potentially contribute to neuroprotection. In particular, Moxon-Lexter suggested that increased perfusion would increase the washout of lactate from hypoxic astrocytes, thereby decreasing intracellular osmolarity and astrocyte swelling. However, direct measurements of vessel diameters near the lesion revealed no significant differences with or without 2-MeSADP treatment (Fig. S7). This data suggest that neither PGE2-mediated vasodilation nor washout of lactate play significant roles in 2-MeSADP enhanced astrocyte protection.

Another striking morphological feature that we frequently observed after photothrombosis was the elongation of astrocytes extending outward from the ischemic core (Fig. S6). This radial polarization is suggestive of fluid movement away from the lesion. As discussed above, astrocytes are known to play a critical role in re-establishing ion and water homeostasis post-ischemia [15], [44]. Naus and co-workers also demonstrated that reduced expression of connexin 43 in astrocytes significantly increased the size of ischemic infarcts and they suggested that gap junctional coupling played a critical role in the removal of ions [45], [46].

Pharmacological and genetic evidence that the protective effect of 2MeSADP is restricted to astrocyte mitochondria was presented in Fig. 6. Ruthenium 360 (Ru_360_) is a relatively specific blocker of mitochondrial Ca^2+^ uptake via the Ca^2+^ uniporter [47]. Importantly, it is not a toxin that blocks or inhibits mitochondrial metabolism. It only affects processes that are dependent on Ca^2+^ uptake into the mitochondria. For neurons, mitochondrial Ca^2+^ rapidly results in necrotic cell death. Consequently, blocking Ca^2+^ uptake with Ruthenium 360 would be expected to be protective if neuronal mitochondria were the target. However, our data show that Ruthenium 360 treatment blocked P2Y_1_R enhanced protection, consistent with an astrocytic receptor target, presumably due to stimulation of Ca^2+^ sensitive matrix dehydrogenases [17], [18]. These data do not rule out an affect of 2MeSADP treatment on vascular or microglia cells, but taken as a whole, the data are more consistent with an astrocytic target and suggest that mitochondrial ATP production plays a rate-limiting role in astrocyte swelling, presumably because of the energy-dependence of ion homeostasis.

It has been reported that 2MeSADP induces platelet aggregation *in vitro* and that this ligand stimulates constriction of blood vessel segments [48]. Both effects would be expected to worsen protection and increase ischemic injury. First, we measured the affect of 2MeSADP treatment on vasodilation, which is normally observed after clot formation, and found no significant difference (Fig. S7, Table S1). Second, 2MeSADP treatment significantly reduced the size of brain infarctions when introduced 24 hours after the initial clot formation. Since trapped Rose-Bengal dye within the clot was still fluorescent, we could verify the stability of the clot at 48 hours (Fig. 3). 2MeSADP has also been reported to have some affinity for the microglial P2Y_12_R, even though it is considered a highly selective ligand for P2Y_1_Rs. However, it seems unlikely that microglia mediate a significant portion of 2MeSADP protection, since disruption of astrocyte mitochondrial function blocks protection. Together, our data suggest that enhanced protection cannot be attributed to affects on platelet aggregation, vasodilation, clot stability or microglia.

Finally, we note that the focal single vessel stroke model induced by RB-photothrombosis differs from the traditional middle cerebral artery occlusion (MCAO) model. The ischemic volume is significantly smaller and the pathophysiology may be more closely related to silent strokes, which are not clinically apparent, but can be observed in magnetic resonance imaging (MRI) studies [49]. The prevalence of silent strokes increases significantly in the elderly and has been correlated with decreases in mental and physical functioning [50]. Irrespective of the pathophysiological correlate, data generated from this study suggest that astrocyte mitochondrial energy metabolism is a critical determinant of the severity of cytotoxic edema and cerebral ischemic infarcts. Enhancement of this energy source is likely to significantly improve the neurological outcome of multiple types of brain injury.

## Materials and Methods

### Animal preparation and surgery

Male FVB/NJ mice (4–6 months), either wildtype or transgenic (FVB/N-Tg(GFAPGFP) 14Mes/J, JAX® Mice, Bar Harbor, ME), were used in this study. Initially, mice were anesthetized at 3% isoflurane with 100% oxygen and subsequently maintained at 1% isoflurane through a nosecone. Depth of anesthesia was carefully monitored and regulated according to vital signs, pinch withdrawal and eye blinks. Body temperature was maintained at 37°C by a feedback-controlled heating pad (Gaymar T/Pump). Vital signs including oxygen saturation, respiratory rate, and heart rate were continuously monitored by using the MouseOx system (STARR Life Sciences). The hair on the mouse head was trimmed and a small incision was made in the scalp to expose the skull. A custom-made stainless steel plate was glued to the skull with VetBond Tissue Adhesive (3M, St Paul, MN). A cranial imaging window, either open-skull or thinned-skull window, was created over the right primary somatosensory cortex (∼1.5 mm posterior to Bregma and 2 mm lateral from midline) depending on the experiment. For the open-skull window, a craniotomy was made using a variable-speed electric drill (Fine Science Tools, Foster City, California, U.S.A.) and the dura matter was carefully removed using a dura hook. Artificial cerebrospinal fluid (aCSF) containing 126 mM NaCl, 2.5 mM KCl, 1.25 mM NaH_2_PO_4_, 2 mM MgCl_2_, 2 mM CaCl_2_, 10 mM glucose and 26 mM NaHCO_3_ (pH 7.4) was maintained on the cortical surface. For the thinned-skull window, a large region of the skull was first thinned with the electric drill and then further thinned with a surgical blade. The final thickness of the thinned skull was approximately 50 µm. Care was taken to avoid any damage to pial vessels and underlying brain tissue. After the cranial imaging window was created, mice were transferred to the microscope stage and used for photothrombosis or imaging experiments. For the repeat imaging experiments, the plate was carefully detached from the skull and the scalp was sutured (Ethicon 6-0 silk suture). After each experiment, the mice were either returned to cages until the next time point or sacrificed. All procedures were approved by the Institutional Animal Care and Use Committee (IACUC) at University of Texas Health Science Center at San Antonio (Animal Welfare Assurance Number: A3345-01).

### Rose Bengal photothrombotic clotting and drug application

Mice were given a 0.1ml tail-vein injection of sterilized Rose Bengal (RB, Sigma, U.S.A.) in artificial cerebral spinal fluid (aCSF). The RB concentration was 20 mg/ml for large lesions (∼300×300 µm^2^) used for Xenogen CD40 imaging and TTC staining experiments, and 10 mg/ml for the single arteriole clotting experiments. Arteries on cortical surface were identified by the blood flow direction and traced downstream to arterioles. A targeted arteriole (20∼30 µm) or cortical region was centered in the imaging field and illuminated with a green laser (543 nm, 5mW) using a 0.8-NA 40× water-immersion objective (Nikon, Tokyo). Clot formation was monitored in real time until the targeted vessel or downstream capillaries were firmly occluded. Stable clots were subsequently identified by a non-fluorescent vessel segmentation ending with highly fluorescent regions. In control experiments, either laser illumination or Rose Bengal itself did not lead to clot formation. 2MeSADP (100 µM, 0.1ml) or fluoroacetate (100 µM, 0.1ml) was induced through tail-vein injections. The treatment concentration of 100 µM for 2MeSADP was used for all experiments reported in this manuscript, since lower concentrations of 2MeSADP (10 µM, 0.1ml) had no significant effect on the lesion size (1.58±0.16, mean ± SD×10^9^ photons, n = 6) compared to control animals (1.66±0.15, n = 3). Higher dose of 2MeSADP (0.1ml, 1000 µM, 2.7 mg/kg) caused respiratory inhibition, convulsion, tremor and seizures after administration.

### *In vivo* Imaging and Analysis

All images were obtained on a confocal microscope initially using an Olympus FV500 that was later upgraded to an FV1000 MPE. As indicated, a 10×0.45 NA dry, 40×0.8 NA or 60×1.1 NA water objective was used. Prism software was used for two-tailed unpaired *t*-test and ANOVA test. The significance level is set at p<0.05. Data are presented as mean ± standard error (SD) of the mean.

### Post photothrombotic infarction evaluation

The size of cerebral infarcts was evaluated 3 ways. First, a membrane protein CD40 was used as an infarction marker [51] and visualized using fluorescently labeled CD40 antibody (Allophycocyanin (APC) tagged anti-mouse CD40, eBioscience) on a Xenogen IVIS 200 fluorescent imaging system (Xenogen Corp, Alameda,CA). A similar non-invasive visualization procedure was recently published by Wunder et al. [52]. Imaging acquisition parameters were set at 1 sec exposure time, small binning, 8 f/stop and 6.5 cm field of view using 615–655 nm excitation and 695–770 nm emission filters. Data were acquired and analyzed with Living Image 2.51 software. APC-anti-mouse CD40 was injected into the tail vein 16 hours prior to imaging. TTC staining was evaluated at 1 and 5 days post-RB-induced-photothrombosis. Mice were sacrificed by cervical dislocation, their brains removed and then placed in ice cold HBSS for 3 minutes. The brain was subsequently transferred to a brain mold (KOPF), sliced into 1 mm sections and immersed in 2% TTC (5 min) at 37°C. The sections were fixed in 10% buffered formaldehyde solution overnight at 4°C. Slices were imaged on a flatbed scanner (HP Scanjet 8300) for analysis of the lesion size at 1200 dpi. The third method of evaluating the infarction was measurement of the extent of astrocyte death, which has previously been suggested as a determinant of the ischemic infarct size [28]. Overlapping z-stack images were periodically obtained around the target arteriole (60 optical sections at 2 µm z-steps) before and after RB-induced photothrombosis as indicated. Three-dimensional z stacks were converted into 2-dimentions using a maximum intensity projection. A montage image of the Z-projection images was constructed using NIH ImageJ software and the MosaicJ plug-in. The infarction border was identified as the absence of GFP fluorescence. Astrocyte swelling was also measured on the 2-dimensional projections of 3-dimensional volumes (maximum intensity pixel projection).

## Supporting Information

Figure S1 Photothrombotic model of cerebral ischemic stroke. (A) A mouse is anesthetized and immobilized with a stainless holders as described in methods. (B) Field of view from a dissecting scope looking down on the mouse cranium. The skull is carefully thinned over a 4–8 mm^2^ region, which permit high resolution confocal imaging. Small white-framed rectangle if approximate field of view in a confocal microscope using a 10× objective. (C) Reconstructed mosaic image of the cortical region from a GFAP-GFP mouse. Rhodamine-dextran was tail-vein injected to illuminate vasculature. Montage was created from ∼30 image stacks (2 µm z-steps). Individual stacks were projected onto a single image using a maximum intensity projection, then montaged together using ImageJ plugin mosaic. (D) Higher magnification single field images obtained with a 40× objective. Lumens of the blood vessels are filled with RB dye. Single red blood cells are apparent as negative (dark) streaks when blood is flowing. A precise single vessel clot (white arrow) is induced at higher zoom (3×) by irradiating with 543 nm for ∼5 minutes. Subsequent panels show development of the clot and absence of blood flow in the final panel, indicated by white arrow.
(1.58 MB TIF)

Figure S2 Photothrombosis breaks down the blood brain barrier (BBB). (A) Sequential high magnification images of the mouse cortex from GFAP-GFP mice prior to (panel 1) and after injection of RB red fluorescent dye (bottom 3 panels). Note that the dye clears within 30 minutes if the vessel is not clotted. (B) Same cortical region after a second bolus of RB was tail-vein injected. Region highlighted in panel 2a with dashed box was irradiated with 543 nm light. After ∼5 minutes, a thrombotic clot formed (indicated by white arrow in top panel). Leakage of RB dye into surrounding astrocytes is detectable within 10 minutes as indicated by white arrows in the bottom 3 panels. (C) Same region of the cortex at lower magnification, 2 days after the initial photothrombosis. RB was tail-vein injected a third time. Dye leakage is again readily apparent in the region surrounding initial clot as indicated by white arrows.
(3.24 MB TIF)

Figure S3 RB-induced lesions in wildtype versus GFAP-tTA-mtEcoRI mice, in the presence of dox, are indistinguishable and reduced by 2MeSADP treatment. (A) Fluorescent images of RB-induced cerebral infarcts of anesthetized wildtype (left panels) and GFATP-tTA-mtEcoRI mice (right panels) on days 2, 3 and 4 after the initial photothrombosis. (B) Line plots of average intensity of the RB-induced infarcts times their area are presented for each group of animals (n = 3 pairs, no significant difference). (C–D) RB-induced cerebral infarcts in GFATP-tTA-mtEcoRI (dox on) mice (right panels) with and without 2MeSADP. Cerebral infarcts were fluorescently labeled with an allophycocyanin (APC)-CD40 antibody 16 hours earlier by tail vein injection. Images were acquired on a Xenogen IVIS 200 fluorescent imaging system.
(1.69 MB TIF)

Figure S4 Decreased levels of mtDNA in dox off GFAP-tTA-mtEcoRI mice is specific to astrocytes. (A and B) Coronal sections (25 µm) of hippocampal CA1 regions from control and GFAP-tTA-mtEcoRI (dox off for 3 weeks) mice immunostained with antibodies specific for neurons (MAP2, red) and astrocytes (GFAP, green) and mitochondrial DNA (white, shown only at higher magnifications). Dashed boxes designate regions of neurons (N1-3) and astrocytes (A1-3) that are presented at higher magnification (5× zoom) in panels C–F as indicated, (C and D) Neuronal regions N1–3 in the molecular layer of the hippocampus with merged image of antibody labeled mtDNA (white). Insets of mtDNA staining from single neurons are presented below each panel. (E and F) Astrocytes A1–3 below the molecular layer with merged images of labeled mtDNA or mtDNA by itself. (G and H) Histograms of the frequency distribution based on the intensity of the mtDNA summed from 6 fields for each mouse. Image J was used to determine intensity levels using the Particle Analyzer tool. Panels are maximum intensity projections of 8 optical sections (2 µm steps), collected with a 40× objective (1.4 NA oil immersion) on a confocal microscope (Olympus FV1000). GraphPad Prism software was used to plot the frequency distribution of both neurons and astrocytes from each mouse (control and GFAP-tTA-mtEcoRI dox off).
(6.78 MB TIF)

Figure S5 Dox off-regulated expression of GFAP-tTA-mtEcoRI decreases the average mitochondrial membrane potential (Δψ) in primary cultures of astrocytes. (A) Confocal images of cultured Astrocytes incubated with Dox (Dox on). Single mitochondria are stained with the potential sensitive dye tetramethyl rhodamine methyl ester (TMRM). (B) Confocal images of mitochondria in astrocytes that have been cultured without Dox (Dox off) for 8 days. (C) Histogram plot of the distribution of Δψ pooled from 12 (dox on) and 15 (dox off) cells is shifted to lower values in a bimodal fashion when Dox is removed. Greater than 250 single mitochondria were analyzed for each group.
(1.46 MB TIF)

Figure S6 Radial polarization of astrocytes after focal single vessel photothrombosis. (A–C) Higher magnification images of the focal cortical lesions presented in Figure. 2A and Figure. 5A, C. White dashed arrow indicates the direction to the center of the blood clot. Image panels to the right are higher magnifications of the regions indicated by the white dashed rectangles.
(3.15 MB TIF)

Figure S7 2-MeSADP treatment does not affect dilation of blood vessels after single vessel photothrombosis. Single blood vessel diameters were measured before and after RB-induced photothrombosis. Average diameters at day 0 were calculated for control and 2MeSADP-treated vessels between 20 and 39 µm, 40 to 59 µm and 60 to 79 µms. Vessel diameters on days 1 and 2 were measured and normalized with their respective day 0 value. The average normalized percent increase times the average day 0 vessel diameter is plotted. Numerical values are presented in Table S1. No significant differences were observed between control and treated.
(0.10 MB TIF)

Table S1 The average vessel diameter of RB induced photothrombosis, in the presence or absence of 2MeSADP (100 µM), on Day 0, 1, and 2 defined by the range of pre-induction of photothrombosis vessel diameter. Average diameters at day 0 were calculated for control and 2MeSADP-treated vessels between 20 and 39 um, 40 to 59 µm and 60 to 79 µms. Vessel diameters on days 1 and 2 were measured and normalized with their respective day 0 value. The average normalized percent increase times the average day 0 vessel diameter is shown in Figure S7.
(0.51 MB DOC)

Movie S1 Astrocyte swelling and oncosis near the boundary of an ischemic core in a live mouse cortex. Confocal images were collected on a single z-plane using a 40× objective. The region being imagined is at the boundary of lysed and swollen cells that define an ischemic core. Astrocytes fluorescence is due to the expression of GFP.
(1.51 MB MOV)

Movie S2 Reversable astrocyte swelling distal to the ischemic core. Confocal images were collected at a single z-plane using a 40× objective. The imaged region shows GFP-expressing astrocytes near a blood vessel. An astrocyte endfoot process initially swells and then shrinks.
(2.01 MB MOV)
