# Supplementary figures and images for: Purinergic Receptor Stimulation Reduces Cytotoxic Edema and Brain Infarcts in Mouse Induced by Photothrombosis by Energizing Glial Mitochondria
Source: PLoS One. 2010 Dec 22;5(12):e14401. doi: 10.1371/journal.pone.0014401 (PMC3008710; doi:10.1371/journal.pone.0014401)

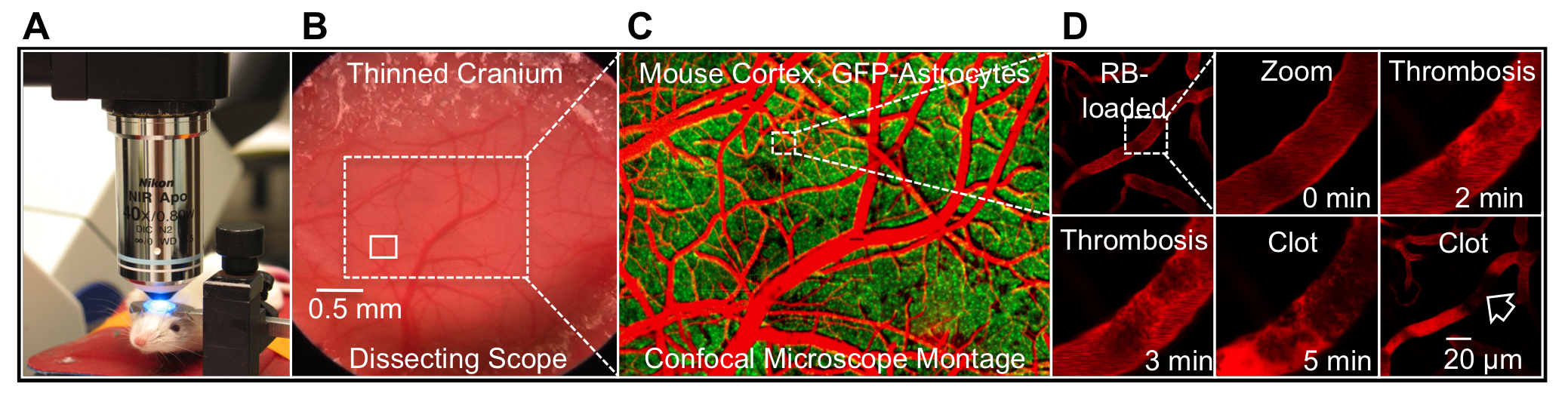

Supplement: Figure S1 — Photothrombotic model of cerebral ischemic stroke. (A) A mouse is anesthetized and immobilized with a stainless holders as described in methods. (B) Field of view from a dissecting scope looking down on the mouse cranium. The skull is carefully thinned over a 4–8 mm2 region, which permit high resolution confocal imaging. Small white-framed rectangle if approximate field of view in a confocal microscope using a 10× objective. (C) Reconstructed mosaic image of the cortical region from a GFAP-GFP mouse. Rhodamine-dextran was tail-vein injected to illuminate vasculature. Montage was created from ∼30 image stacks (2 µm z-steps). Individual stacks were projected onto a single image using a maximum intensity projection, then montaged together using ImageJ plugin mosaic. (D) Higher magnification single field images obtained with a 40× objective. Lumens of the blood vessels are filled with RB dye. Single red blood cells are apparent as negative (dark) streaks when blood is flowing. A precise single vessel clot (white arrow) is induced at higher zoom (3×) by irradiating with 543 nm for ∼5 minutes. Subsequent panels show development of the clot and absence of blood flow in the final panel, indicated by white arrow. (1.58 MB TIF) [file pone.0014401.s001.tif]

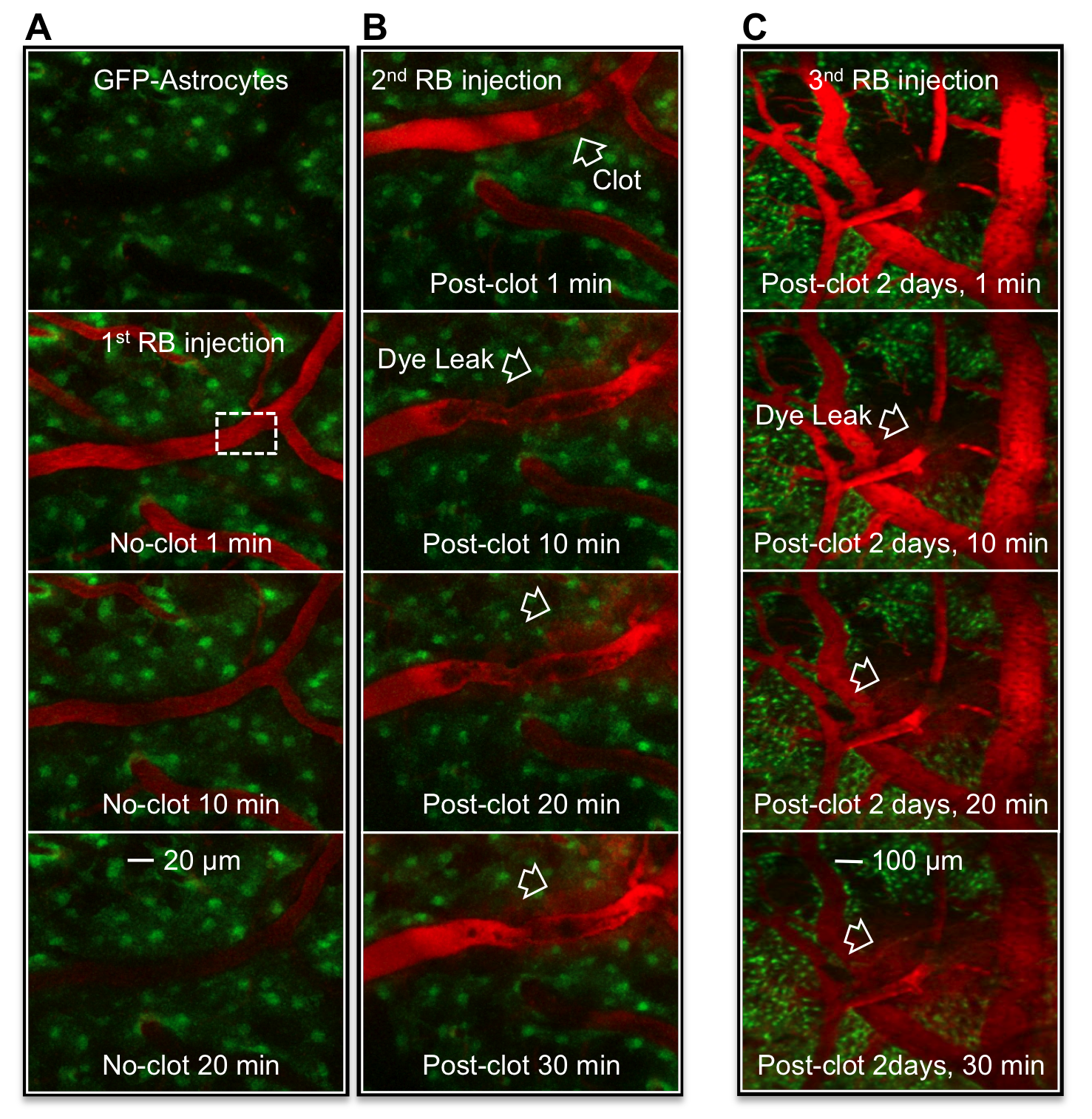

Supplement: Figure S2 — Photothrombosis breaks down the blood brain barrier (BBB). (A) Sequential high magnification images of the mouse cortex from GFAP-GFP mice prior to (panel 1) and after injection of RB red fluorescent dye (bottom 3 panels). Note that the dye clears within 30 minutes if the vessel is not clotted. (B) Same cortical region after a second bolus of RB was tail-vein injected. Region highlighted in panel 2a with dashed box was irradiated with 543 nm light. After ∼5 minutes, a thrombotic clot formed (indicated by white arrow in top panel). Leakage of RB dye into surrounding astrocytes is detectable within 10 minutes as indicated by white arrows in the bottom 3 panels. (C) Same region of the cortex at lower magnification, 2 days after the initial photothrombosis. RB was tail-vein injected a third time. Dye leakage is again readily apparent in the region surrounding initial clot as indicated by white arrows. (3.24 MB TIF) [file pone.0014401.s002.tif]

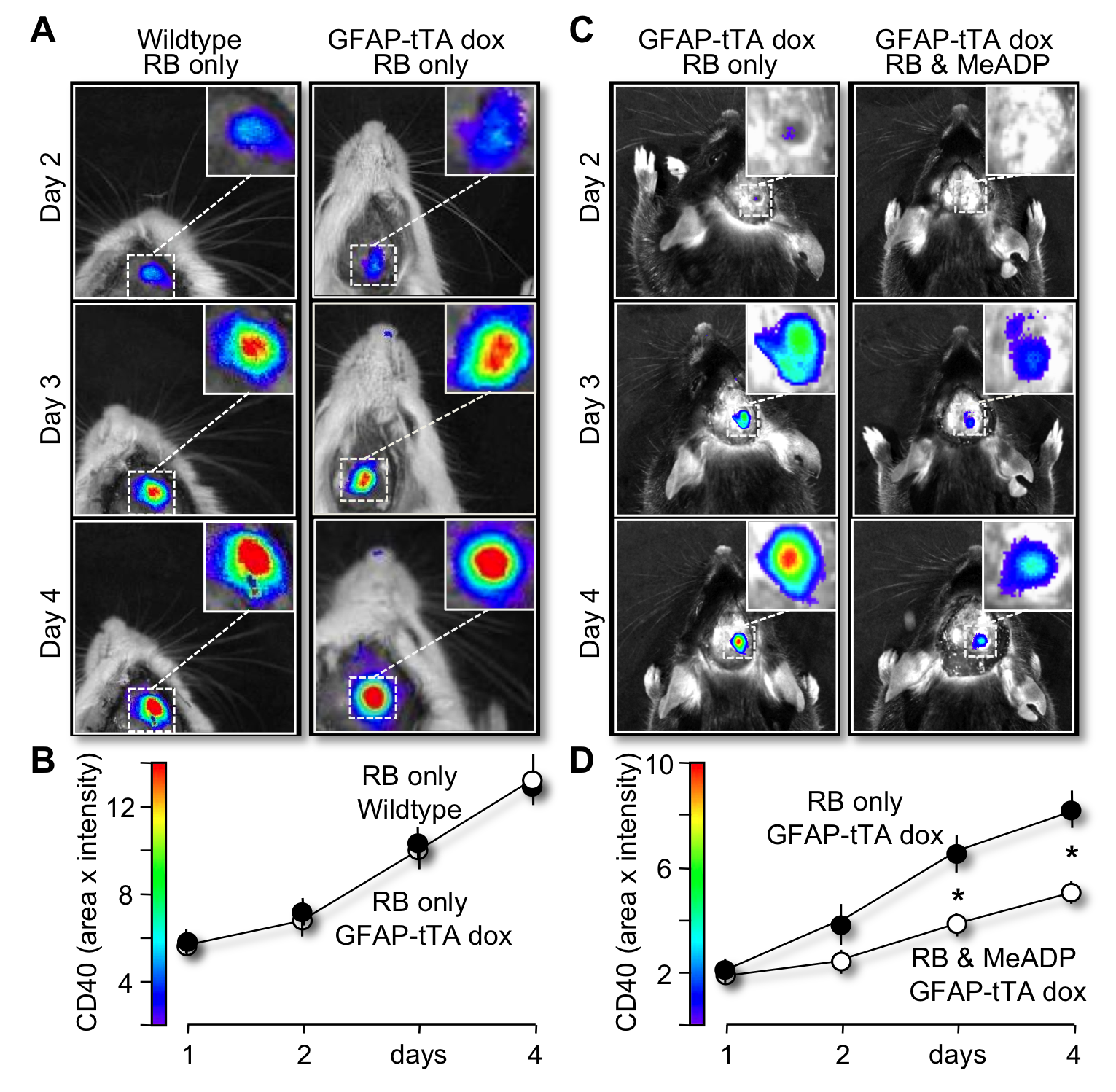

Supplement: Figure S3 — RB-induced lesions in wildtype versus GFAP-tTA-mtEcoRI mice, in the presence of dox, are indistinguishable and reduced by 2MeSADP treatment. (A) Fluorescent images of RB-induced cerebral infarcts of anesthetized wildtype (left panels) and GFATP-tTA-mtEcoRI mice (right panels) on days 2, 3 and 4 after the initial photothrombosis. (B) Line plots of average intensity of the RB-induced infarcts times their area are presented for each group of animals (n = 3 pairs, no significant difference). (C–D) RB-induced cerebral infarcts in GFATP-tTA-mtEcoRI (dox on) mice (right panels) with and without 2MeSADP. Cerebral infarcts were fluorescently labeled with an allophycocyanin (APC)-CD40 antibody 16 hours earlier by tail vein injection. Images were acquired on a Xenogen IVIS 200 fluorescent imaging system. (1.69 MB TIF) [file pone.0014401.s003.tif]

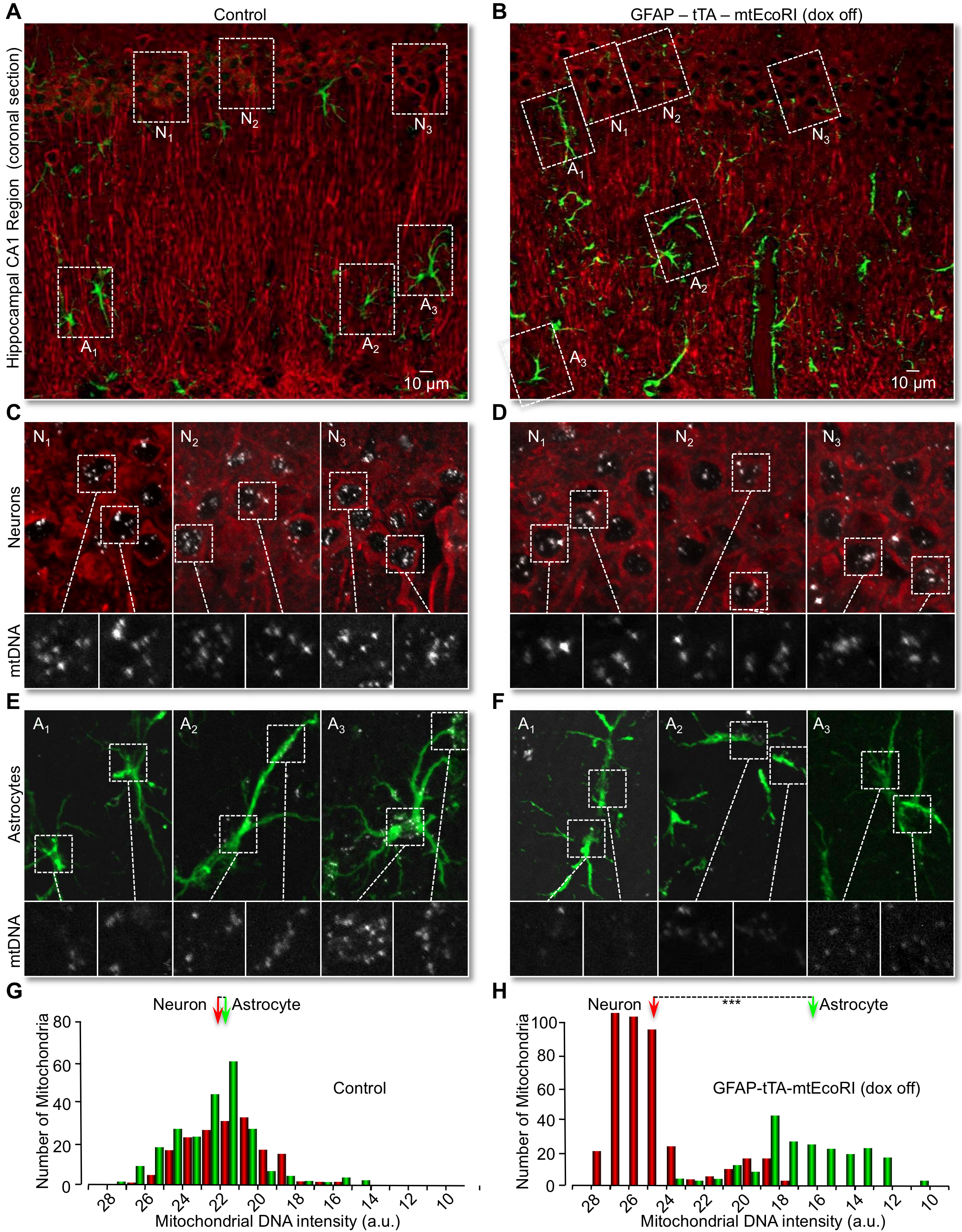

Supplement: Figure S4 — Decreased levels of mtDNA in dox off GFAP-tTA-mtEcoRI mice is specific to astrocytes. (A and B) Coronal sections (25 µm) of hippocampal CA1 regions from control and GFAP-tTA-mtEcoRI (dox off for 3 weeks) mice immunostained with antibodies specific for neurons (MAP2, red) and astrocytes (GFAP, green) and mitochondrial DNA (white, shown only at higher magnifications). Dashed boxes designate regions of neurons (N1-3) and astrocytes (A1-3) that are presented at higher magnification (5× zoom) in panels C–F as indicated, (C and D) Neuronal regions N1–3 in the molecular layer of the hippocampus with merged image of antibody labeled mtDNA (white). Insets of mtDNA staining from single neurons are presented below each panel. (E and F) Astrocytes A1–3 below the molecular layer with merged images of labeled mtDNA or mtDNA by itself. (G and H) Histograms of the frequency distribution based on the intensity of the mtDNA summed from 6 fields for each mouse. Image J was used to determine intensity levels using the Particle Analyzer tool. Panels are maximum intensity projections of 8 optical sections (2 µm steps), collected with a 40× objective (1.4 NA oil immersion) on a confocal microscope (Olympus FV1000). GraphPad Prism software was used to plot the frequency distribution of both neurons and astrocytes from each mouse (control and GFAP-tTA-mtEcoRI dox off). (6.78 MB TIF) [file pone.0014401.s004.tif]

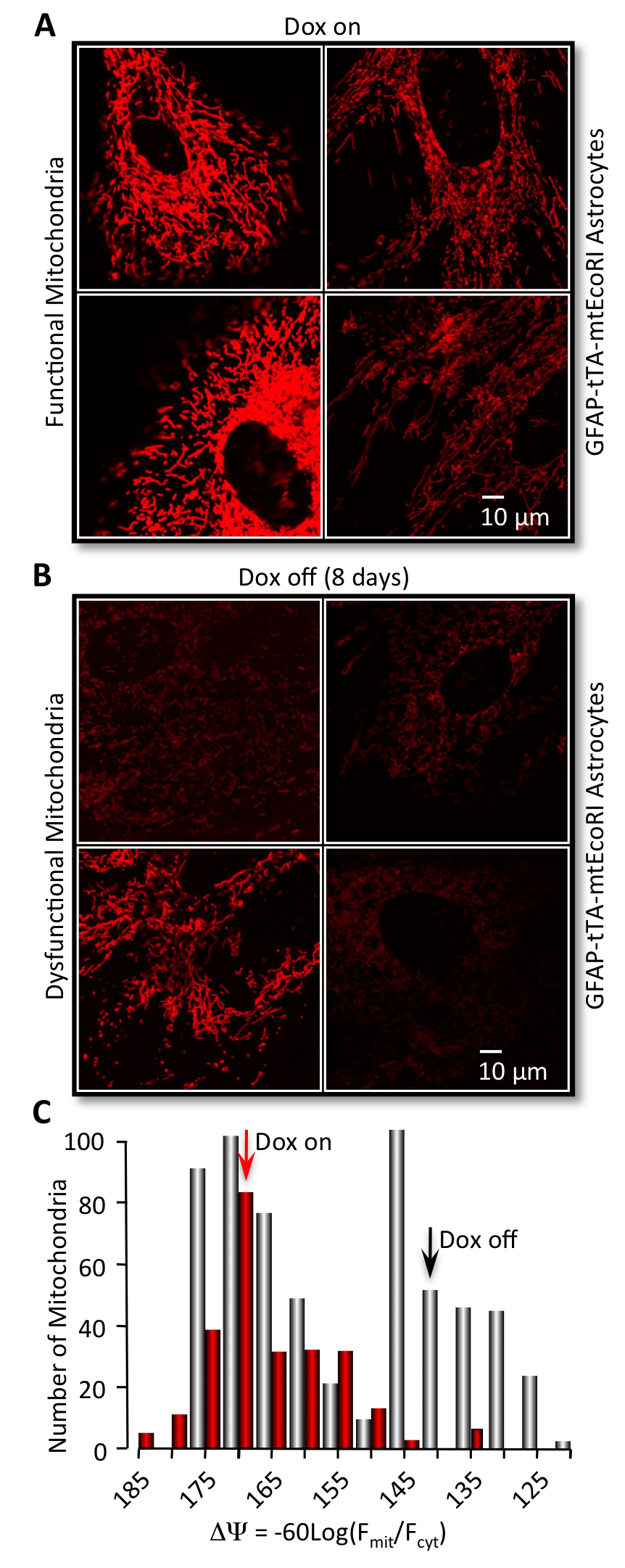

Supplement: Figure S5 — Dox off-regulated expression of GFAP-tTA-mtEcoRI decreases the average mitochondrial membrane potential (Δψ) in primary cultures of astrocytes. (A) Confocal images of cultured Astrocytes incubated with Dox (Dox on). Single mitochondria are stained with the potential sensitive dye tetramethyl rhodamine methyl ester (TMRM). (B) Confocal images of mitochondria in astrocytes that have been cultured without Dox (Dox off) for 8 days. (C) Histogram plot of the distribution of Δψ pooled from 12 (dox on) and 15 (dox off) cells is shifted to lower values in a bimodal fashion when Dox is removed. Greater than 250 single mitochondria were analyzed for each group. (1.46 MB TIF) [file pone.0014401.s005.tif]

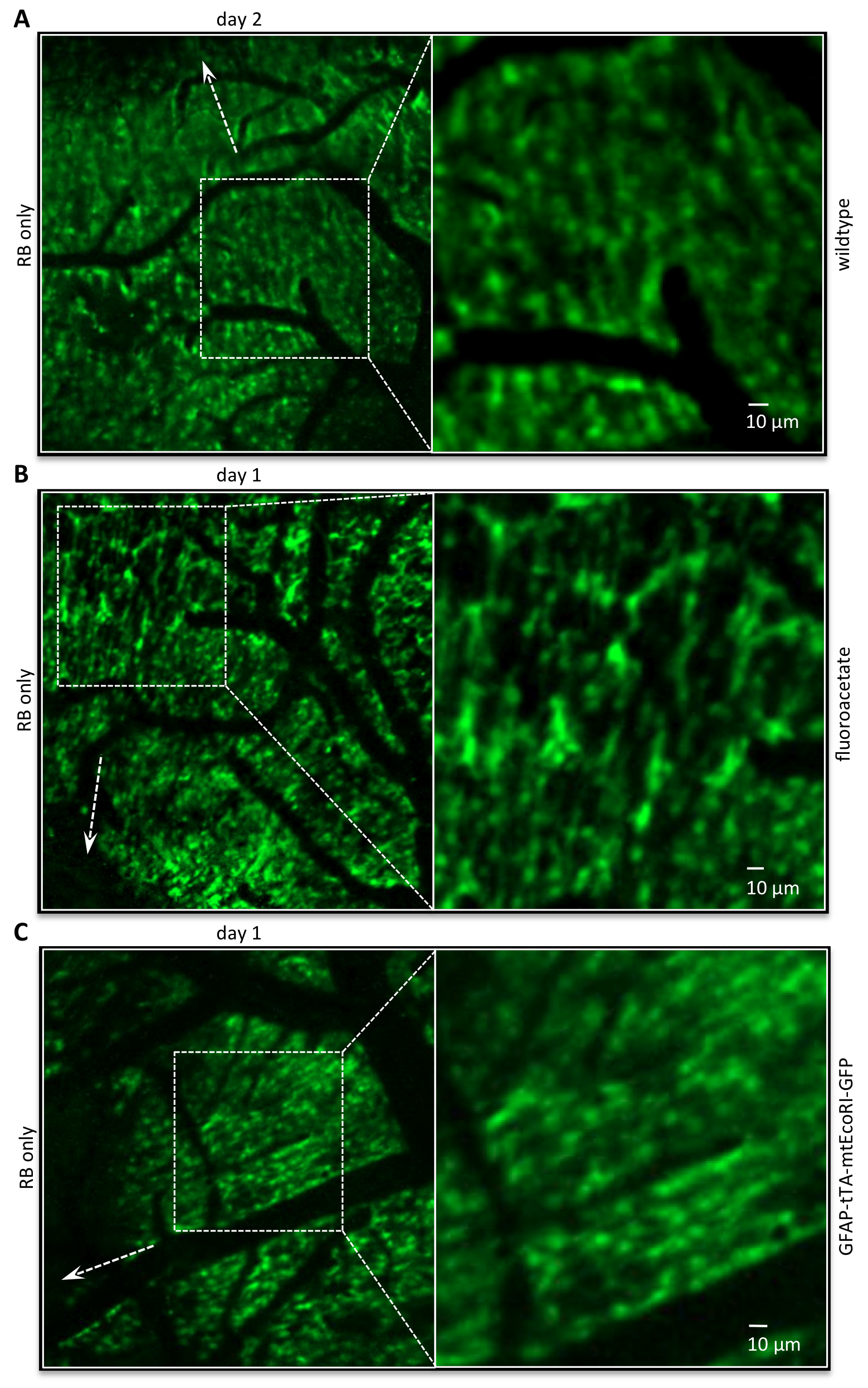

Supplement: Figure S6 — Radial polarization of astrocytes after focal single vessel photothrombosis. (A–C) Higher magnification images of the focal cortical lesions presented in Figure. 2A and Figure. 5A, C. White dashed arrow indicates the direction to the center of the blood clot. Image panels to the right are higher magnifications of the regions indicated by the white dashed rectangles. (3.15 MB TIF) [file pone.0014401.s006.tif]

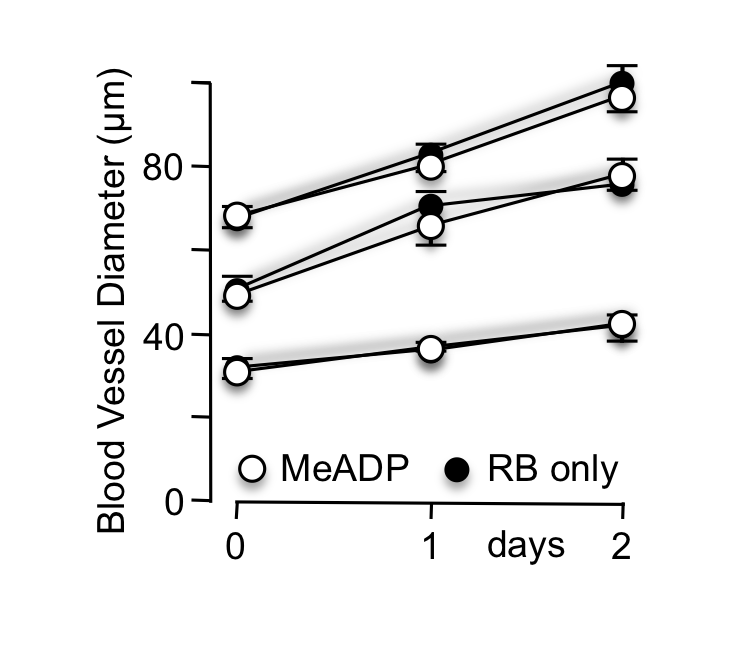

Supplement: Figure S7 — 2-MeSADP treatment does not affect dilation of blood vessels after single vessel photothrombosis. Single blood vessel diameters were measured before and after RB-induced photothrombosis. Average diameters at day 0 were calculated for control and 2MeSADP-treated vessels between 20 and 39 µm, 40 to 59 µm and 60 to 79 µms. Vessel diameters on days 1 and 2 were measured and normalized with their respective day 0 value. The average normalized percent increase times the average day 0 vessel diameter is plotted. Numerical values are presented in Table S1. No significant differences were observed between control and treated. (0.10 MB TIF) [file pone.0014401.s007.tif]

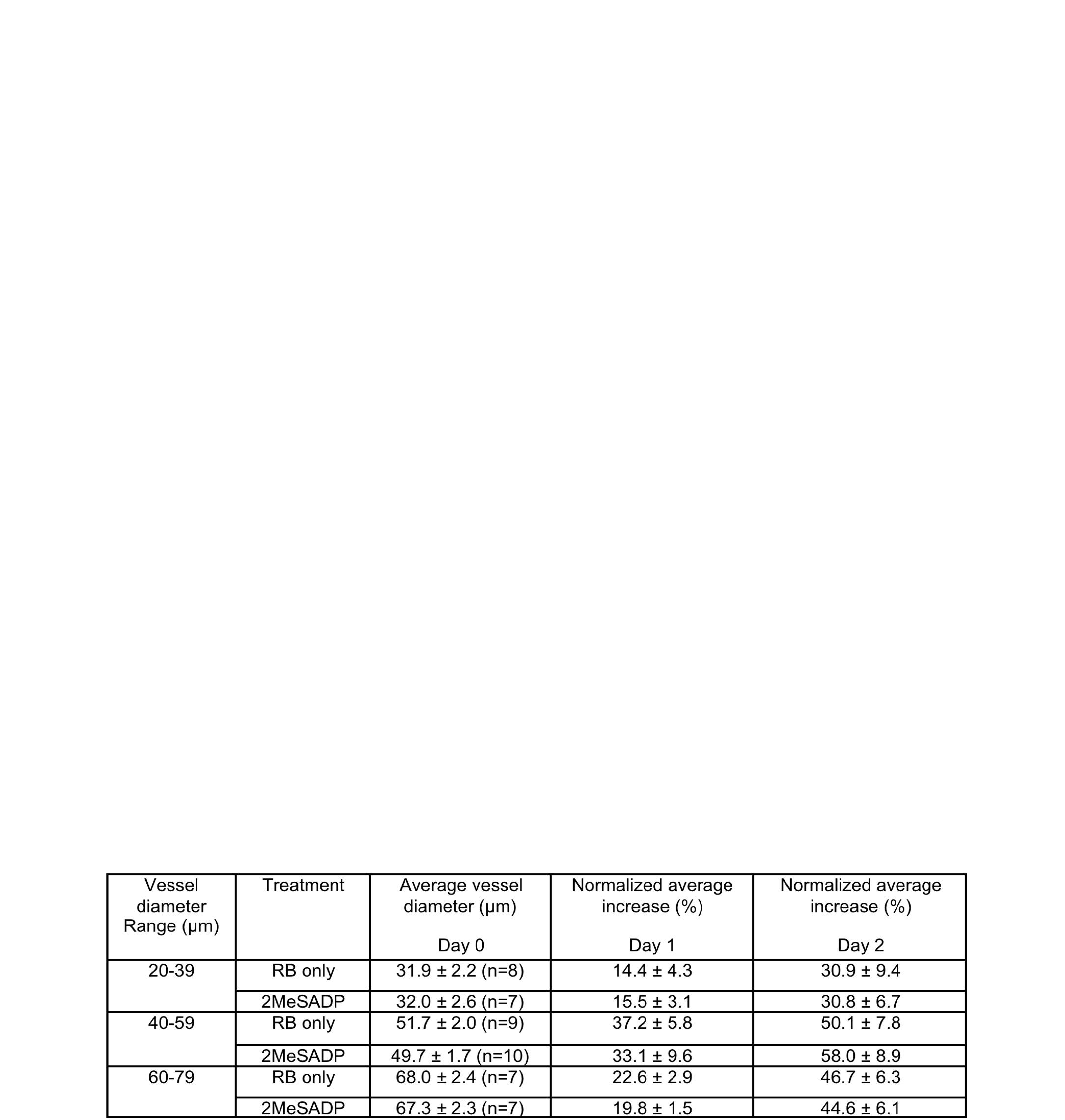

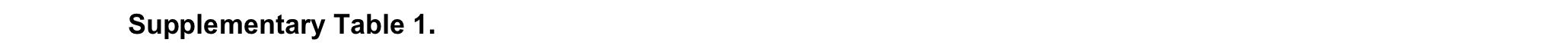

Supplement: Table S1 — The average vessel diameter of RB induced photothrombosis, in the presence or absence of 2MeSADP (100 µM), on Day 0, 1, and 2 defined by the range of pre-induction of photothrombosis vessel diameter. Average diameters at day 0 were calculated for control and 2MeSADP-treated vessels between 20 and 39 um, 40 to 59 µm and 60 to 79 µms. Vessel diameters on days 1 and 2 were measured and normalized with their respective day 0 value. The average normalized percent increase times the average day 0 vessel diameter is shown in Figure S7. (0.51 MB DOC) [file pone.0014401.s008.doc]
